# Predictive accuracy of particle filtering in dynamic models supporting outbreak projections

**DOI:** 10.1186/s12879-017-2726-9

**Published:** 2017-09-26

**Authors:** Anahita Safarishahrbijari, Aydin Teyhouee, Cheryl Waldner, Juxin Liu, Nathaniel D. Osgood

**Affiliations:** 1 Department of Computer Science, University of Saskatchewan, 176 Thorvaldson Building, 110 Science Place, Saskatoon, SK - S7N5C9 Canada; 2 Western College of Veterinary Medicine, University of Saskatchewan, Campus Drive, Saskatoon, Canada; 3 Department of Mathematics and Statistics, University of Saskatchewan, College Drive, Saskatoon, Canada

**Keywords:** Particle filtering, System dynamics, Transmission model, Compartmental model, Stochastic, Outbreaks, Infectious diseases, Communicable illness, Empirical observations

## Abstract

**Background:**

While a new generation of computational statistics algorithms and availability of data streams raises the potential for recurrently regrounding dynamic models with incoming observations, the effectiveness of such arrangements can be highly subject to specifics of the configuration (e.g., frequency of sampling and representation of behaviour change), and there has been little attempt to identify effective configurations.

**Methods:**

Combining dynamic models with particle filtering, we explored a solution focusing on creating quickly formulated models regrounded automatically and recurrently as new data becomes available. Given a latent underlying case count, we assumed that observed incident case counts followed a negative binomial distribution. In accordance with the condensation algorithm, each such observation led to updating of particle weights. We evaluated the effectiveness of various particle filtering configurations against each other and against an approach without particle filtering according to the accuracy of the model in predicting future prevalence, given data to a certain point and a norm-based discrepancy metric. We examined the effectiveness of particle filtering under varying times between observations, negative binomial dispersion parameters, and rates with which the contact rate could evolve.

**Results:**

We observed that more frequent observations of empirical data yielded super-linearly improved accuracy in model predictions. We further found that for the data studied here, the most favourable assumptions to make regarding the parameters associated with the negative binomial distribution and changes in contact rate were robust across observation frequency and the observation point in the outbreak.

**Conclusion:**

Combining dynamic models with particle filtering can perform well in projecting future evolution of an outbreak. Most importantly, the remarkable improvements in predictive accuracy resulting from more frequent sampling suggest that investments to achieve efficient reporting mechanisms may be more than paid back by improved planning capacity. The robustness of the results on particle filter configuration in this case study suggests that it may be possible to formulate effective standard guidelines and regularized approaches for such techniques in particular epidemiological contexts. Most importantly, the work tentatively suggests potential for health decision makers to secure strong guidance when anticipating outbreak evolution for emerging infectious diseases by combining even very rough models with particle filtering method.

## Background

According to World Health Organization (WHO), seasonal influenza viruses cause 3 to 5 million cases of severe illness, with about 250,000 to 500,000 deaths each year, with emerging-strains sometimes significantly increasing this burden. An important example of this was high-burden emergence of pandemic influenza A (H1N1) during the 2009–2010 influenza season. Vaccination and intervention strategies such as school closures for early mitigation of pandemic influenza spread may reduce severe complications and deaths [1]. Key concerns during an outbreak include staffing requirements for implementation of a pandemic response, clinical resource constraints [2], managing individuals’ expectations and behaviors, which often relate their risk perception [3], and mobilization of health resources [4]. Rapid or ideally real-time reporting of surveillance data provide a clear picture of what has happened, but fail to provide clarity on how the epidemic will evolve. Simulation modeling can be an important tool to anticipate what is most likely to happen in the near future, to ask questions concerning interventions and identify desirable policies.

Mathematical models describing the dynamic of epidemiological infections can be useful for projection purposes [5–9], but often the fundamental challenge in leveraging models for emerging communicable diseases and strains is that there is limited epidemiological knowledge regarding the natural history of infection and the values needed for model parameters [10]. While a well-formulated model can be useful for planning, often the knowledge needed to build that model is lacking at the time when it is the most urgently needed. In this situation, a precisely calibrated and highly tuned model can play an important role, but is often infeasible to build in a time compatible with planning needs. Even for models of endemic infections such as seasonal influenza in which refined estimates of parameter values and understanding of natural history are available, model predictions secured early in an outbreak inevitably diverge from observations [11–13]. This reflects the fact that all models are simplifications (and thus inevitably omit factors). In addition, stochastics are involved in real-world systems, which depend on unpredictable or hard-to-predict factors such as shifting vaccine attitudes and risk perception that can impact contact patterns [14–16], as well as the vagaries of transmission and the health system response. This divergence is made more likely by the fact that many such factors—including changes in human contact patterns—are believed to play a substantial role in disease transmissions [15–17] and are often not captured in models. Statistical filtering and estimation methods for dynamic models, such as Sequential Monte Carlo (SMC) and Markov Chain Monte Carlo (MCMC) methods, provide an attractive tool to not only create model predictions based on where we are right now, but to use empirical observations from continuing surveillance to reground that model on an ongoing basis [12, 18–22].

Among estimation algorithms, Kalman filtering is a long and heavily used tool for creating estimates based on consensus of empirical data and model predictions using Maximum Likelihood Estimation (MLE) [23–27]. However, it is hampered by stiff distributional assumptions regarding process and measurement error. The Kalman filter’s reliance on gaussian assumption and MLE further limits its accuracy, particularly in the context of non-linear systems. The reliance of Kalman filtering on linearization of nonlinear distributions both raises strong challenges for accurate state estimation in the context of infrequent observations and limits the applicability of such models to an important but circumscribed subset of transmission models for which linearization is possible [28].

As a SMC, particle filtering offers similar overall types of benefits as Kalman filtering while relaxing such constraints. Particle filtering deals with less restrictive assumptions concerning the noise and process model, and samples from a joint distribution of state trajectories rather than conforming to a MLE approach. This method [29] samples from the posterior distribution of model state trajectories, combining empirical data and model dynamics. Key mechanics of particle filtering are drawn from the “importance sampling” method. With importance sampling, we sample from a particular distribution from which sampling is difficult (target distribution) in a two-phased approach in which we first draw weighted samples from an alternative distribution (importance proposal distribution) that retains the major properties of the target distribution, and then sample from those weighted samples with a probability proportional to their weight. Similar to importance sampling, in a particle filter, sampling is performed from the particles based on their weights. When new empirical data arrive, the filter further updates the weights to reflect the fitness of particles to these observations (as quantified by the ratio of the target distribution to the proposal distribution). The method that we use here to update the weight of particles is based on the “condensation algorithm” [30, 31], in which the weight of each particle is updated at each observation time by multiplying it by the likelihood of observing the observed data given the state of that particle at that point in time. Following [32], and our previous success in applying this approach for previous transmission models [28, 33, 34], we assume that the likelihood distribution is characterized by a negative binomial distribution:

1 $$  P(y_{t}|i_{t}) =\dbinom {y_{t}+r-1}{y_{t}}p^{y_{t}}(1-p)^{r}  $$

where $p=\frac {i_{t}}{i_{t}+r}$, *r* is a dispersion parameter, *y*_*t*_ is the model observation (number of incident cases reported for time t), and *i*_*t*_ is the incident case count recorded over a scenario-specific interval.

The objective of this study was to apply particle filtering to predictive models of emerging communicable diseases, which are often built in the presence of limited information about underlying parameters. In light of the growing availability of epidemiological data streams, we seek here to investigate the impact on model accuracy of varying the inter-observation interval, studying the tradeoff between pursuing more frequent but more noisy sampling and less frequent but more stable estimates. We further examine the robustness of the particle filter to different assumptions concerning behaviour change and assumptions regarding observational error.

## Methods

We formulated a transmission model for an influenza-like disease in a classic compartmental fashion and used it with the SMC method of particle filtering.

**Fig. 1:**
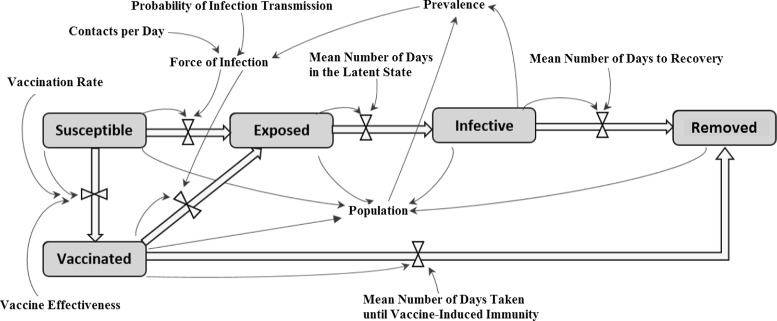
Transmission model

The dynamic model includes Susceptible (S), Exposed (E), Infective (I), Removed (R), and Vaccinated (V) stocks (Fig. 1). It bears noting that the Vaccinated state represents a transient set of individuals who have received the vaccine but have not yet attained immunity; upon achieving immunity, such individuals transition to the Removed state. The aggregate compartmental state equations describing the model stocks are given as follows:

2 $$ \dot{S}= -c\beta\frac {I}{S+E+I+R+V}S-abS  $$

3 $$ \begin{aligned} \dot{E} &= c\beta\frac {I}{S+E+I+R+V}S\\ &\quad+ c\beta\frac {I}{S+E+I+R+V}V - \frac{E}{\tau} \end{aligned}  $$

4 $$ \dot{I} = \frac{E}{\tau}-\frac{I}{\mu}  $$

5 $$ \dot{R} = \frac{I}{\mu}+\frac{V}{v_{a}}  $$

6 $$ \dot{V} = abS-\frac{V}{v_{a}}-c\beta\frac{I}{S+E+I+R+V}V  $$

where *c*, *β*, *τ*, *μ*, *v*_*a*_, *a* and *b* represent contacts per day, probability of infection transmission given exposure, mean number of days in the latent state, mean number of days to recovery, mean number of days for immunity to develop, per capita vaccination rate and vaccine effectiveness parameter, respectively. Vaccine delivery rates were obtained from public data made available by Manitoba Health, Healthy Living and Seniors for the second wave of pandemic H1N1 and for the period October 6th, 2009 through to January 4th, 2010.

In our model, each particle is associated with a complete copy of model state, including the state of two evolving parameters of the model: contact rate (*c*) and fraction of reported incidents (*f*)- *fI* accounts for fractional actual reporting-, which are associated with evolving state variables whose values can be sampled by particle filtering. Thus, each particle is associated with a vector of model states [*S, E, I, R, V, c, f*]. Following [28, 32], a negative binomial distribution is assumed to link the observed incident case count for a specified time period to the underlying count of individuals emerging from latency in the model. We preferred a negative binomial distribution over the binomial distribution due to the robustness of negative binomial distribution for the particle filtering methodology [28]. It particularly avoids the risk of a situation in which all particles are associated with zero weights, causing a singularity during weight renormalization. As the model runs and learns from the empirical data over time, the particles associated with the stocks that exhibit the greatest fitness - in terms of explaining the observed data - survive, are replicated and henceforth evolve independently.

This work builds on previous work by Osgood and Liu evaluating particle filtering against ground truth from an agent-based model [28] and our previous work evaluating particle filtering in terms of its ability to predict future reported real world prevalence in the absence of a ground truth model [33]. In this work, we seek to examine the impact on model predictive accuracy of the inter-observation interval of empirical data, and the robustness of ranges of plausible values for the dispersion parameter and the parameters associated with the random walk associated with *c* and *f*. Such variations are examined for a number of different observation points during the outbreak.

The prediction of particle filtering was evaluated against empirical data publicly available from Manitoba Health, Healthy Living and Seniors, which included daily confirmed cases of pandemic H1N1 for the period of October 6th, 2009 through January 4th, 2010. To judge the deviation of particle filtering prediction from observations, we defined the discrepancy metric as the expected value of the *L* ^2^ norm of the difference between sampled particles. By sampling *n* particles (*n*=1000), the discrepancy value was obtained using the following equation:

7 $$  discrepancy= \frac{\sum_{i=T^{*}+1}^{T_{f}} \left(\frac{\sum_{j=1}^{n}\left(x_{ij}^{P}-x_{i}^{E}\right)^{2}}{n}\right)}{T_{f}-T^{*}}  $$

where $x_{ij}^{P}$ is the expected sample associated with sampled particle *j* at observation *i*, $x_{i}^{E}$ is the respective empirical data at observation *i*. *T*_*f*_ is the end time being set equal to 91 and *T* ^∗^ indicated the time *t* up to which the particles’ weights were updated based on observation, where 0≤*t*≤*T* ^∗^. In other words, the data before and equal to this time was taken into account for particle filtering based on the observed data; after time *T* ^∗^, particle weights were no longer updated using the empirical data, no further resampling occurred, and we evaluated how well particle filtering predicted the remaining empirical data.

### Parameter values

#### Initial values

**Fig. 2:**
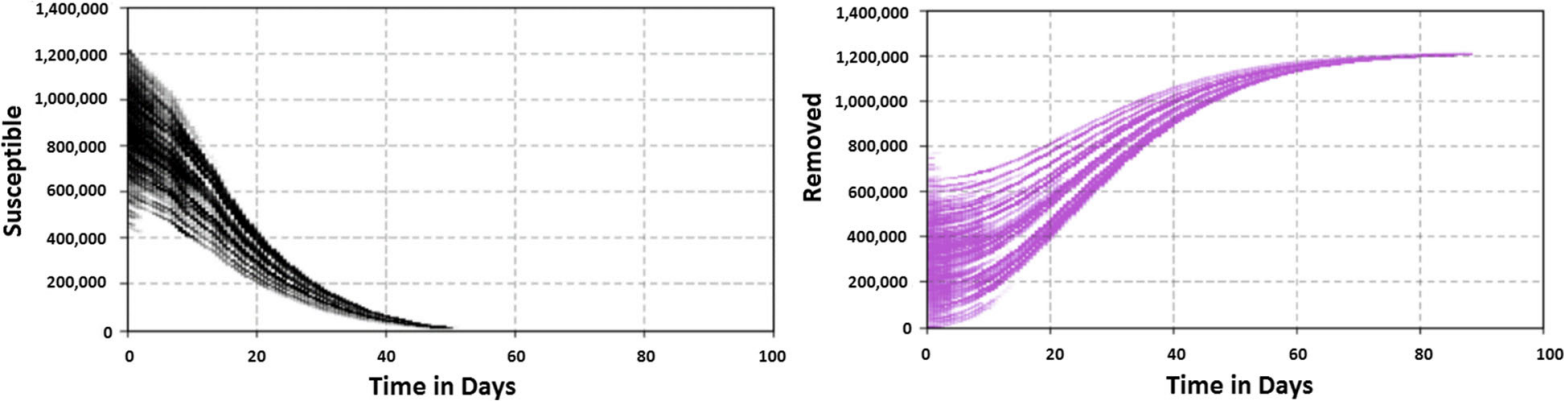
Progress of susceptible and removed stocks over time, initializing with a range of values

We set the initial value of Susceptible and Removed stocks based on sampling from a truncated normal distribution instead of considering the initial values as a static number. Figure 2 gives curves for Susceptible and Removed stocks. Detailed information about initial values is provided in Appendix A.

#### Contacts per unit time (*c*)

In this work, particle filtering contributes to the estimation of this dynamic parameter over time through particle selection. This parameter - which carries a non-negative value - is log transformed, with the logarithm evolving according to an (unbounded) zero-mean gaussian random walk with standard deviation (*γ*) (8). High values of *γ* allow the contact rate to evolve more quickly, while low values of *γ* would be associated with assumptions of comparatively slow changes in contact rate. In this work, we examined model behavior over a wide range of *γ* to identify appropriate ranges for this important parameter. The initial value of the stock associated with the logarithm of *c* is set to the logarithm of the uniform distribution on the interval between minimum contacts per day and maximum contacts per day which have been considered as 1 and 300, respectively (9).

8 $$ \frac{d(\text{log}\ c)}{dt}= N\left(0,\gamma^{2}\right)  $$

9 $$ (\text{log}\ c)|_{t=t_{0}} = \text{ln}\ (\text{U} (c_{min}, c_{max}))  $$

#### Fraction reported incidence

The other stochastic parameter included here represents the fraction of reported incidents (*f*). The fraction of people who present for care (and are reported to public health authorities) when emerging from the latent state is an uncertain value. It is also likely to evolve according to risk perception on the part of the population and provider perception of the importance of reporting. As for *c*, we considered (a transformed value of) this parameter as a state of the model and thus associated each particle with a value for this parameter. We considered the transformed version of this parameter as evolving according to a zero-mean gaussian random walk with a standard deviation given by a parameter (*η*). Since *f* is a fraction varying between 0 and 1, the (unbounded) random walk was conducted on the logit of this parameter (10) - which was itself the aspect of model state - and the initial value of this state is set to the logit of fraction reported incidence sampled from a continuous uniform distribution on the interval between 0 and 1 (11).

10 $$ \frac{d(\text{logit}\ f)}{dt}= N\left(0,\eta^{2}\right)  $$

11 $$ (\text{logit}\ f)|_{t=t_{0}} = \text{logit}\ (\text{U} (0, 1))  $$

The other parameters of the model are considered as static and are shown in Table 1.

**Table 1:** Table showing parameters

Parameter name	Notation	Value	Source	Units
Probability of infection transmission given exposure	*β*	0.06	Expert opinion	Unit
Mean time to recovery	*μ*	7	[38]	Day
Vaccine effectiveness	*b*	0.9	[39]	Unit
Mean time taken for antibodies to develop	*v*_*a*_	14	Expert opinion	Day
Total population size	*N*	1214403	[40]	Person
Mean latent time	*τ*	Uniformly distributed (2, 4)	[38]	Day
Vaccination rate	*a*	Extracted from empirical vaccinated percentage		1/Day

## Scenarios

We formulated a set of scenarios to explore how the error associated with particle filtered model predictions would respond to changes in the total period for which empirical data was available to the model (*T* ^∗^), the frequency of and degree of aggregation associated with empirical data observations supplied to the model, contact rate volatility parameter (*γ*) and dispersion parameter (*r*).

### Adequacy of empirical data (*T*^∗^)

We examined the impact of particle filter on model predictive accuracy at various time points during the progression of an outbreak. This simulated a situation in which a health authority is partway through an outbreak and can only take into account data observed until this point when making predictions for coming weeks. Specifically, in each scenario, particle filtering used data from the start of the outbreak up to and equal to a time *T* ^∗^; the accuracy of particle filter was then evaluated in predicting the data for all times after *T* ^∗^. We considered *T* ^∗^ equal to 35, 42, 49, and 56, equivalent to predictions made at 5, 6, 7 and 8 weeks into the outbreak.

### Inter-observation aggregation interval/frequency of data observations

Based on the existence of noise in the clinically observed data, there is a trade-off between employing more frequently observed (but less aggregate) data and reducing the noise associated with each data point via observations that are aggregated over longer periods of time. Employing more frequent sampling - by using shorter time intervals between observations - yields more numerous data points, but each such datum will typically exhibit greater proportional variability. By contrast, employing less frequent sampling during training (thereby aggregating data over a longer period between observations) leads to fewer but proportionately less noisy individual data points. To examine the impact of the frequency of data observations on filtered model accuracy, we investigated the impact of aggregating empirical data used in particle filtering observations at three levels. First, we considered daily data - i.e., the number of people clinically confirmed as infected per day - to update the particles weights during particle filtering. Because the original data source specifies data on a daily basis, no further aggregation was required for this case. Second, data was aggregated over three days for the purposes of particle filtering. In the third and final alternative setting, the particle filtering used data aggregated on a weekly basis. It should be emphasized that such aggregation affected only the model observations, and not the calculation of discrepancies between model results and empirical data.

### Random walk standard deviation parameter (*γ*)

To explore the changes in contact per unit time patterns during an outbreak, and its effect on the spread of infection, we performed particle filtering using alternative values for the contact rate variability parameter (*γ*). In order to explore a broad dynamic range, we examined parameter values at successive powers of two of the smallest value: 0.125, 0.25, 0.5, 1, 2, 4 and 8.

### Dispersion parameter (*r*)

The ability of particle filtering to project incident case counts is sensitive to the dispersion parameter value associated with the negative binomial distribution. Increasing the dispersion parameter makes the negative binomial distribution tighter, while retaining the same mean value [35]. We compared the discrepancy resulting from running the model with alternative values of the dispersion parameter to developing an understanding as to how this parameter affects predictive accuracy. To ensure the comparability of scenarios when running the models using three-day and weekly observations, we considered the *r* parameter respectively three times and seven times as great as the *r* that we used when observing daily data. This linear scaling of the dispersion parameter *r* with sampling period reflects the fact that as the inter-observation interval rises, the likelihood function is operating with observed values for incident case counts that are correspondingly larger, and the resulting dispersion would also be expected to scale in the same way. To identify the way in which model discrepancy changes with the dispersion parameter, and to identify the dispersion parameter that offers the greatest accuracy, we ran scenarios considering different values of this parameter. Values 1, 2, 4, 8, 16 and 32 were examined for experiments regarding the daily scenario, while values 3, 6, 12, 24, 48 and 96 were used for three-day experiments and values 7, 14, 28, 56, 112 and 224 were used for weekly experiments.

### Statistical analysis discrepancy results

To provide an objective assessment of the differences in discrepancy associated with each of the variables considered in the above scenarios, we employed Box-Cox multivariable regression analysis [36]. Box-Cox analysis was selected rather than traditional multiple linear regression as the discrepancy results were not normally distributed and routinely used transformations did not adequately address the assumptions of normality or homogeneous variance. The adequacy of empirical data (*T* ^∗^), inter-observation interval or frequency of data observations, contact rate random walk standard deviation parameter (*γ*), and dispersion parameter (*r*) were evaluated as categorical variables as none of the parameters appeared to have a linear association with discrepancy based on data visualization exercises and there was also interest in understanding the specific differences among the chosen parameter values. Differences with *p* values < 0.05 were considered statistically significant.

## Results

**Fig. 3:**
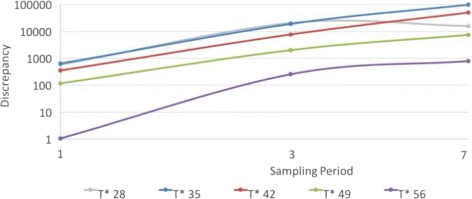
Log of discrepancy vs. log of sampling period for different observation times (*r*=32, *γ*= 0.125)

On the basis of running the model using daily, accumulated three days and accumulated weekly empirical data, particle filtering observing daily data performed consistently and markedly better than while observing three-day and weekly data. Particle filtering using successively larger sampling periods yielded super-linearly higher levels of discrepancy (Fig. 3, Tables 2, 3 and 4). The exact difference in discrepancy between sampling periods varies by the amount of data available (as given by *T* ^∗^), but consistently the discrepancy extending from particle filtering using daily data was orders of magnitude smaller than for the larger sampling periods. Tables showing the discrepancy of particle filtering predictions in frequency scenarios for different observation times and *γ*=0.125 and *γ*=2 are included in Appendix B. The observed super-linear scaling of error with inter-observation interval was similar when comparing three day vs. weekly sampling.

**Table 2:** Discrepancy of particle filtering predictions in frequency scenarios for different observation times and *γ*=0.25

Frequency scenarios (*γ*=0.25)	*T* ^∗^=35	*T* ^∗^=42	*T* ^∗^=49	*T* ^∗^=56
PF using daily data, *r*=2	380	225	69	0
PF using three-day data, *r*=6	11453	5667	1646	205
PF using weekly data, *r*=14	80850	39578	6291	482
PF using daily data, *r*=8	384	213	29	0
PF using three-day data, *r*=24	14044	6452	1249	79
PF using weekly data, *r*=56	104043	42248	5484	447
PF using daily data, *r*=32	230	196	45	0
PF using three-day data, *r*=96	13617	4701	1096	86
PF using weekly data, *r*=224	149164	39232	4945	250

**Table 3:** Discrepancy of particle filtering predictions in frequency scenarios for different observation times and *γ*=0.5

Frequency scenarios (*γ*=0.5)	*T* ^∗^=35	*T* ^∗^=42	*T* ^∗^=49	*T* ^∗^=56
PF using daily data, *r*=2	474	270	80	0
PF using three-day data, *r*=6	13038	6577	1637	128
PF using weekly data, *r*=14	97325	38652	6661	592
PF using daily data, *r*=8	337	230	66	0
PF using three-day data, *r*=24	14900	6482	1264	67
PF using weekly data, *r*=56	126163	43288	5761	418
PF using daily data, *r*=32	635	188	13	0
PF using three-day data, *r*=96	13868	4590	766	44
PF using weekly data, *r*=224	156099	45808	4231	277

**Table 4:** Discrepancy of particle filtering predictions in frequency scenarios for different observation times and *γ*=1

Frequency scenarios (*γ*=1)	*T* ^∗^=35	*T* ^∗^=42	*T* ^∗^=49	*T* ^∗^=56
PF using daily data, *r*=2	3327	695	87	0
PF using three-day data, *r*=6	43931	12590	1630	39
PF using weekly data, *r*=14	645037	154916	16362	976
PF using daily data, *r*=8	1568	241	18	0
PF using three-day data, *r*=24	35024	6251	682	4
PF using weekly data, *r*=56	1216215	129467	6072	376
PF using daily data, *r*=32	904	104	5	0
PF using three-day data, *r*=96	25452	4199	393	0
PF using weekly data, *r*=224	1243398	129629	4580	254

After accounting for differences across all of the examined scenarios for the adequacy of empirical data (*T* ^∗^), random walk standard deviation parameter (*γ*), and dispersion parameter (*r*), the average discrepancy was significantly greater for data collected over three-day (*p*<0.001) and seven-day (*p*<0.001) intervals than for daily data.

**Fig. 4:**
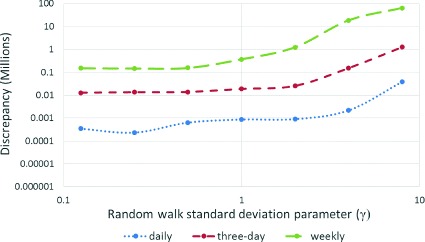
Discrepancy versus random walk standard deviation using daily, three-day and weekly observations (*T* ^∗^=35 and *r*=32 for daily, 96 for three-day, and 224 for weekly observations)

**Fig. 5:**
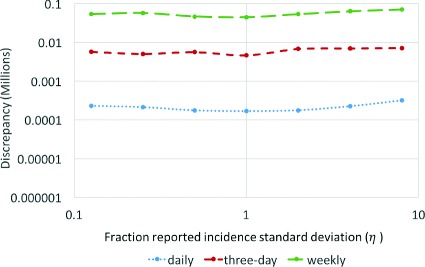
Discrepancy in terms of dispersion parameter and random walk standard deviation - daily empirical data and *T* ^∗^=42

**Fig. 6:**
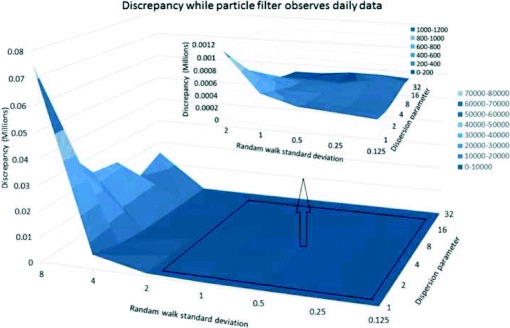
Discrepancy in terms of dispersion parameter and random walk standard deviation - empirical data available every three-days and *T* ^∗^=42

**Fig. 7:**
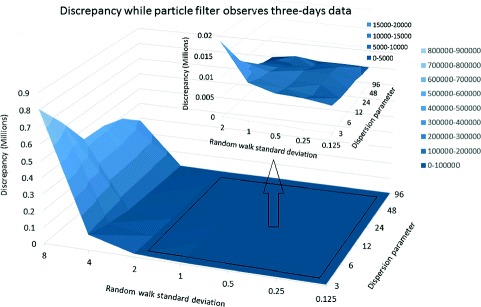
Discrepancy in terms of dispersion parameter and random walk standard deviation - weekly empirical data and *T* ^∗^=42

The effect of the standard deviation for the random walk in the log of the contact rate (*γ*) also exhibited pronounced scaling patterns. Plotting three dimensional surfaces to represent the change of discrepancy in terms of this parameter *γ* and dispersion parameter *r*, we observed that for all daily, every-three-day and weekly scenarios, a *γ* parameter in the range of 0 to 2 yields markedly reduced discrepancy compared with *γ* values above 2 (Figs. 4, 5, 6 and 7). After accounting for differences across all of the examined scenarios for the frequency of data collection, adequacy of empirical data (*T* ^∗^), and dispersion parameter (*r*), the average discrepancy was significantly greater for random walk standard deviation values of 4 (*p*<0.001) and 8 (*p*<0.001) compared to the baseline value of 0.125. However, there was no significant difference between random walk standard deviation values of 0.25 (*p*=0.97), 0.5 (*p*=0.99), 1 (*p*=0.97), or 2 (*p*=0.42) and the baseline random walk standard deviation of 0.125.

**Fig. 8:**
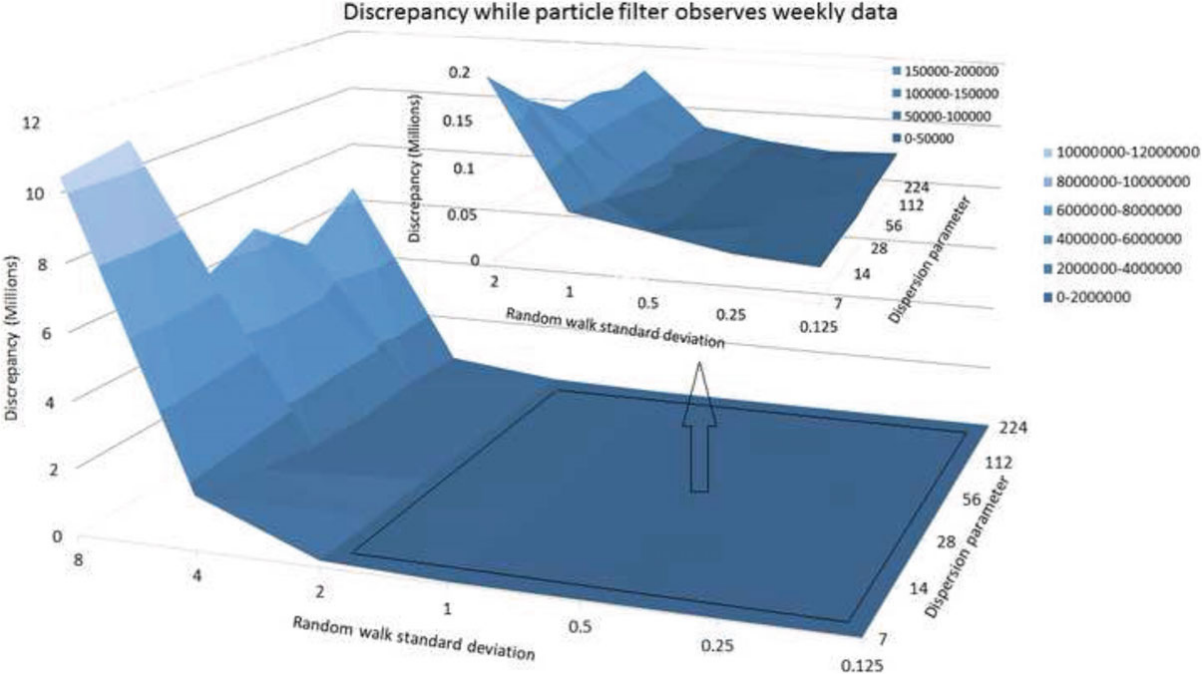
Discrepancy versus fraction reported incidence standard deviation using daily, three-day and weekly observations (*T* ^∗^=35, *γ*=0.125 and *r*=32 for daily, 96 for three-day, and 224 for weekly observations)

Figure 8 presents the discrepancies from particle filtering for different values of standard deviation associated with fraction reported incidence parameter (*η*). It appears that particle filtering behaves robustly to changes in *η* for daily, every-three-day and weekly scenarios. The value for *η* was set to 1 for all of the scenarios reported in this work.

**Fig. 9:**
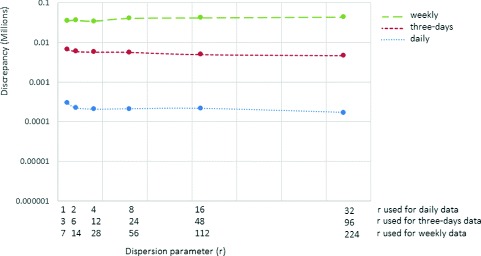
Discrepancy versus dispersion parameter using daily, three-day and weekly observations (*T* ^∗^=42 and *γ*=0.125)

**Fig. 10:**
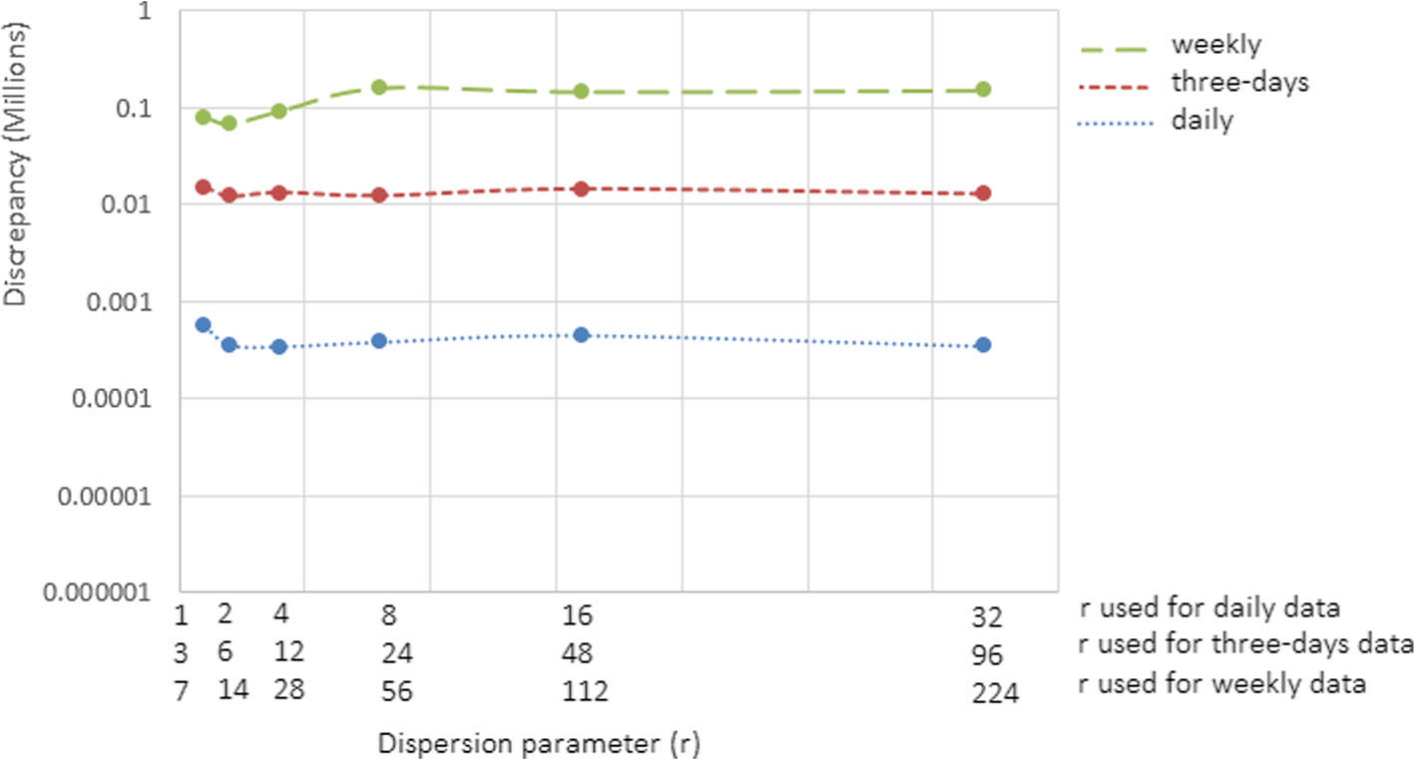
Discrepancy versus dispersion parameter using daily, three-day and weekly observations (*T* ^∗^=35 and *γ*=0.125)

As shown in Figs. 9 and 10, results suggest that increasing the dispersion parameter does not appear to strongly affect the performance of particle filtering at smaller values of contact rate random walk standard deviation parameter (*γ*). However, at larger values of *γ*, the impact of the dispersion parameter become more apparent (Figs. 5, 6 and 7). After accounting for differences across all of the examined scenarios for the frequency of data collection, adequacy of empirical data (*T* ^∗^), and the contact rate random walk standard deviation parameter (*γ*), the average discrepancy was significantly smaller for each increasing dispersion parameter (*r*) from 1 to 32 (*p*<0.001) as compared to the baseline value of 1.

Table 5 shows the discrepancy for the model without particle filtering. The discrepancy for particle filtering scenarios was found to be less than the discrepancy associated with the model without particle filtering.

**Table 5:** Discrepancy without particle filtering in frequency scenarios

Frequency scenarios	Discrepancy
Without PF using daily data	101942842
Without PF using three-day data	386532229
Without PF using weekly data	575977188

## Discussion and future work

The particle filtering method explored here offers considerable potential. The value offered by this approach seems likely to be particularly pronounced when used in the context of emerging communicable diseases in which limited parameter information is available to inform available models, but where frequent (e.g., daily) reporting of case counts are available. Particle filtering supports an adaptive response updating the current state and stochastic parameter values involved in dynamic models. In this way, the models are kept current with the latest evidence, which can be used to predict forward and to be used to then anticipate possible trade-offs between interventions. The key finding in this work is that particle filtering can perform orders of magnitude more accurately in case the daily clinical reports are available. For public health authorities seeking to employ accurate projection systems for communicable disease outbreaks, this finding suggests a premium on putting in place efficient reporting schemes.

A second set of findings relates to the high robustness of preferred particle filtering parameter assumptions as we change the observation time in the outbreak and the inter-observation interval. While the assumption made for dispersion parameter associated with the negative binomial likelihood formulation does exert some impact on the accuracy of particle filtering, the results are far less sensitive to variations in this parameter beyond an inter-observation interval specific threshold. By contrast, while the results are highly sensitive to the assumptions regarding the rate of potential evolution of contacts per unit time (*γ*), the findings across different inter-observation intervals and time of observation are consistent in suggesting a specific range of low values for this parameter. While the particulars of these values are likely to differ somewhat for distinct epidemiological contexts (e.g., pathogens), populations and types of data, the consistency of these results suggests the potential for simpler guidelines to govern the application of particle filtering in specific epidemiological contexts. Importantly, given this robustness and daily reporting, these results suggest favorable starting assumptions for application of this approach to similar pathogens in developed countries. For different epidemiological contexts, the robustness of the results also suggest that a much simpler variant of the methodology used here might be applied in the opening days and weeks of an outbreak to estimate favorable parameter values for the dispersion parameter and rate of contact rate evolution for that particular context.

Research progress is needed to adequately realize particle filtering on other types of models, including agent-based and discrete-event models [37]. Since these modeling techniques are widely used in public health, and since implementing particle filtering in the presence of these types of models is not as straightforward due to software limitations, advances are urgently required to improve software support for particle filtering for such models.

## Conclusion

The findings presented here demonstrate that in the presence of simple models, particle filtering in combination with dynamic models can develop accurate predictive systems in the context of emerging communicable diseases, particularly when models lack information about parameters, but frequent reporting of empirical data is available. The results suggest that more frequent sampling improves predictive accuracy remarkably. The robustness of particle filtering in this case study also suggests that it may be possible to apply a variant of the method presented here to estimate unknown parameters of an emerging outbreak – specifically a new pathogen that is not well-known – in its opening days and weeks. According to the findings in this work, even very rough models can be combined with particle filtering to project the evolution of emerging infectious diseases and secure strong guidance for health policy makers.

## Appendix A: Detailed information about initial values of compartmental states

*S*_0_: Truncated normal distribution, Mean = 900000, Standard deviation = 150000, Lower bound = 0, Upper bound =*N*−*I*_0_, Sample size = number of particles = 10000

- *E*_0_: 0 for all particles
- *I*_0_: 7 for all particles
- *R*_0_: *N* - *S*_0_−*E*_0_−*I*_0_−*V*_0_
- *V*_0_: 0 for all particles

In this model, V class refers to those receiving vaccination during the pandemic (ongoing vaccination). Those being vaccinated prior to the second wave might be part of R class or S depending on vaccine efficacy. Since the initial values of R and S were unclear, we considered the initial values of these states as distributions.

## Appendix B: The discrepancy of particle filtering predictions in frequency scenarios for different observation times and *γ*=0.125 and *γ*=2

**Table 6:** Discrepancy of particle filtering predictions in frequency scenarios for different observation times and *γ*=0.125

Frequency scenarios (*γ*=0.125)	*T* ^∗^=35	*T* ^∗^=42	*T* ^∗^=49	*T* ^∗^=56
PF using daily data, *r*=2	354	225	71	0
PF using three-day data, *r*=6	12109	5945	1593	181
PF using weekly data, *r*=14	68381	36313	6322	608
PF using daily data, *r*=8	381	210	44	0
PF using three-day data, *r*=24	12273	5655	1309	93
PF using weekly data, *r*=56	162378	40820	5670	476
PF using daily data, *r*=32	455	169	13	0
PF using three-day data, *r*=96	12808	4647	1125	90
PF using weekly data, *r*=224	153010	44106	5224	295

**Table 7:** Discrepancy of particle filtering predictions in frequency scenarios for different observation times and *γ*=2.0

Frequency scenarios (*γ*=2.0)	*T* ^∗^=35	*T* ^∗^=42	*T* ^∗^=49	*T* ^∗^=56
PF using daily data, *r*=2	3327	695	87	0
PF using three-day data, *r*=6	43931	12590	1630	39
PF using weekly data, *r*=14	645037	154916	16362	976
PF using daily data, *r*=8	1568	241	18	0
PF using three-day data, *r*=24	35024	6251	682	4
PF using weekly data, *r*=56	1216215	129467	6072	376
PF using daily data, *r*=32	904	104	5	0
PF using three-day data, *r*=96	25452	4199	393	0
PF using weekly data, *r*=224	1243398	129629	4580	254

## Abbreviations

E: Exposed
H1N1: Influenza A
I: Infective
MCMC: Markov Chain Monte Carlo
MLE: Maximum likelihood estimation
R: Removed
S: Susceptible
SMC: Sequential Monte Carlo
V: Vaccinated
WHO: The World Health Organization
